# Diagnosis and treatment of autoimmune retinopathy: review of current approaches

**DOI:** 10.1007/s10792-025-03696-y

**Published:** 2025-08-18

**Authors:** Dimitrios Kalogeropoulos, Andrew John Lotery, Carlos Pavesio, Chris Kalogeropoulos, Panagiotis Kanavaros, Farid Afshar, Fatima Shawkat, Gabriella De Salvo

**Affiliations:** 1 Southampton Eye Unit, University Hospital Southampton, 103 Tremona Rd, Southampton, SO16 6HU UK; 2 Faculty of Medicine, University of Southampton, Southampton, UK; 3 National Institute for Health Research Biomedical Research Centre, Moorfields Eye Hospital, London, UK; 4 UCL-Institute of Ophthalmology, London, UK; 5 Department of Ophthalmology, Faculty of Medicine, School of Health Sciences, University of Ioannina, Ioannina, Greece; 6 Department of Anatomy-Histology-Embryology, Faculty of Medicine, School of Health Sciences, University of Ioannina, Ioannina, Greece

**Keywords:** Autoimmune retinopathy, Cancer-associated retinopathy, Melanoma-associated retinopathy, Anti-retinal autoantibodies, Autoimmune diseases, Malignancies, Masquerades

## Abstract

**Purpose:**

To provide a comprehensive overview of the existing understanding regarding the clinical characteristics, diagnostic investigation, and treatment strategies for autoimmune retinopathy (AIR). To emphasize the lack of consensus in the field and the ongoing controversies regarding best practices.

**Methods:**

Narrative review of the literature on PubMed and Google Scholar databases.

**Results:**

AIR comprises a group of rare autoimmune disorders causing retinal degeneration, characterized by rapid vision deterioration linked to circulating anti-retinal autoantibodies (ARAs). The spectrum includes primary autoimmune diseases and associations with retinitis pigmentosa or various malignancies, raising questions about the causative role of antibodies. Non-paraneoplastic AIR patients may be younger on average than paraneoplastic AIR patients and may have a higher likelihood of being female and having a history of autoimmune disease. Diagnosing AIR is challenging due to its complex pathophysiology, overlapping phenotypes, the absence of standardized diagnostic criteria, and the limited availability of specialized serological testing for ARAs. Despite decades of research progress, the exact mechanisms underlying ocular immune privilege breakdown and the autoimmune attack on retinal cells in AIR remain unknown.

**Conclusions:**

The diagnosis and management of AIR present ongoing challenges, with no international consensus on diagnostic criteria or treatment protocols. While several authors consider the identification of circulating ARAs crucial for diagnosis, there is disagreement on the pathogenicity of specific antibodies, including anti-recoverin. The lack of randomized controlled trials and a universally accepted treatment protocol for AIR further contribute to uncertainties in its management. Despite recent advances, AIR remains an enigmatic condition, necessitating further research to establish standardized diagnostic and therapeutic guidelines.

## Introduction

Autoimmune retinopathy (AIR) refers to a cluster of infrequent autoimmune disorders that lead to retinal degeneration [1]. The AIR spectrum encompasses various pathologies with similar clinical and immunological features. These clinical entities are typically defined by the rapid and progressive deterioration of vision linked to the presence of circulating anti-retinal autoantibodies (ARAs). AIR can manifest either as a primary autoimmune disease or in conjunction with retinitis pigmentosa (RP) or various malignancies [2]. This remains a subject of debate, as it is still unclear whether the presence of antibodies serves as a causative factor or arises as a consequential outcome in the context of the issue at hand. It is imperative to ascertain the pathogenicity of these antibodies. The complex pathophysiology and the overlapping phenotypes of AIR, together with the lack of standardized diagnostic criteria and conclusive examinations make the diagnosis of AIR extremely challenging. Globally, the availability of specialized serological testing of ARAs for clinical purposes is significantly restricted, further complicating the clinical management of patients suspected of having AIR. Despite decades of experimental studies that have led to significant progress in comprehending the pathophysiological mechanisms that may cause AIR, the exact factors responsible for the breakdown of ocular immune privilege and the sudden autoimmune attack on retinal cells remain unknown [1, 2]. This review aims to present a thorough overview of the current state of knowledge, including both established findings and ongoing controversies in AIR.

## Methods

A comprehensive literature search was conducted using the PubMed database, supplemented by Google Scholar. The search strategy included the keywords: "autoimmune retinopathy," "cancer-associated retinopathy," "melanoma-associated retinopathy," and "antiretinal autoantibodies." Relevant articles published up to July 2024 were included. Studies were excluded if they lacked significant information or methodological rigor. Each selected study underwent critical appraisal, assessing study design, sample size, methodology, and potential bias, to ensure a thorough and transparent review process.

## Classification

AIR can be categorized into paraneoplastic (pAIR) and non-paraneoplastic (npAIR) [1, 3, 4]**.** pAIR comprises cancer-associated retinopathy (CAR) and melanoma-associated retinopathy (MAR). npAIR is the form of AIR occurring in the absence of malignancy and is diagnosed by ruling out other possible etiologies. In this review, we explore the three main categories of Autoimmune Retinopathy (AIR): CAR, MAR, and npAIR [5]. The term AIR is employed broadly in this analysis to encompass all three clinical subtypes. It's important to note, however, that this study does not delve into AIR or retinal deterioration stemming from factors like retinitis pigmentosa, ocular trauma, white dot syndromes, paraneoplastic conditions primarily impacting the retinal pigment epithelium (RPE), such as bilateral diffuse uveal melanocytic proliferation (BDUMP), and conditions affecting the optic nerve.

## Historical perspective

In 1976, Sawyer et al. [6] reported the first CAR cases; three females with bronchial carcinoma who developed vision loss due to photoreceptor degeneration. A few years later, immunohistochemistry detected ARAstargeting ganglion cells and photoreceptors in CAR patients [7, 8]. In 1987, a23-kDa retinal antigen targeted by antibodies in the serum of CAR individuals was discovered by Thirkill et al. [3]**.** This retinal antigen was later identified as recoverin. The term "paraneoplastic retinopathy" was introduced by Klingele et al. [9] in 1984. The first case of MAR was described by Gass [10] in 1984 and was later classified as a paraneoplastic retinopathy [11]. It was not before 1997, that the first cases of AIR were recorded in individuals without an underlying neoplasia, exhibiting clinical features similar to CAR [12]. Since then, numerous cases of both pAIR and npAIR have been reported. However, despite significant progress over the past four decades, diagnosing and managing AIR remains challenging, as there is no standardization of diagnostic criteria and ARA testing methods. The majority of research and evidence regarding AIR diagnosis and management has focused on the paraneoplastic form.

## Epidemiology

AIR is an extremely rare condition, accounting for less than 1% of all cases managed at tertiary referral centers [13]. There are currently no available reports on the prevalence of pAIR or npAIR in the literature. The elusive nature of the disease and the lack of established diagnostic criteria may have led to underdiagnosis and misclassification. The understanding of npAIR's natural history, diagnostic, and therapeutic approach largely relies on the pAIR literature, case reports, and expert consensus [14, 15]. A mean age of 55 years (range 18–88 years) was found at diagnosis in a review of 209 patients with CAR and MAR, with a higher prevalence in women than men [16]. The major cancer associations observed included lung (16%), breast (16%), melanoma (16%), hematological (15% comprising of lymphomas, leukemias, and myelomas), gynecological (9%), prostate (7%), and colon (6%) [16]. In fewer than 4% of the cases, AIR presented before a cancer diagnosis. The latency between cancer diagnosis and the onset of retinopathy varied from weeks to months in patients with lung cancers and lymphomas, while it took years in patients with breast and prostate cancers [16]. Rapidly progressive visual loss was associated with a shorter latency between cancer diagnosis and the onset of retinopathy. Anti-recoverin, anti-enolase, anti-carbonic anhydrase II (anti-CAII), and anti-transducin autoantibodies were found in some patients with CAR months to years before cancer diagnosis [16]**.** Adamus et al. [17] found that patients clinically diagnosed with AIR (57.5%, 111/193), including those who were later found to be seronegative for ARAs, were more likely to be females. Furthermore, clinically diagnosed npAIR patients (mean age 55.9 years) were younger than pAIR patients (mean age 62.0 years) on average [17]. Ferreyra et al. [15] reported on 24 consecutive npAIR individuals who were all seropositive for ARAs. The median age was 47 years (range, 11–78 years), and 62.5% (15/24) were women. Additionally, 66.7% (16/24) had a history of autoimmune disorder, and most frequently hypothyroidism [18]. Likewise, an unpublished retrospective case series of 24 seropositive npAIR patients at the National Eye Institute demonstrated that the median age was 51.5 years (range, 37–88 years), 79.2% were female, and 45.8% had a history of autoimmune disease [19].

## Pathophysiology

Retinopathy encompasses damage to the retina arising from inflammatory, infectious, vascular, or degenerative processes, frequently associated with systemic diseases. Among these, diabetic retinopathy stands out as one of the most prevalent conditions [20, 21]. In recent times, researchers have directed their efforts towards AIR [13], a rare and poorly comprehended disorder wherein the immune system mistakenly targets and damages healthy retinal cells, leading to vision impairment [22]. Although the presence of ARAs in the patients' serum is confirmed, their precise role remains uncertain. It is likely that these ARAs are produced due to an overly aggressive immune response to antigens in the retina.

The prevalence of autoimmune diseases in the general population is approximately 7–9%, with a higher occurrence in women than men [23]. An essential objective in autoimmune disease diagnosis is to determine the involvement of ARAs in the pathogenic processes. Autoantigens typically consist of self-proteins or molecules naturally present in the body and recognized by the immune system as part of the "self." However, in certain instances, the immune system may erroneously perceive autoantigens as foreign, triggering an autoimmune response and causing the destruction of healthy tissues, such as retinal tissue in the case of AIR. A key aspect in understanding AIRs lies in the close connection between the retina and thymus [24, 25]. The retina contains numerous proteins expressed also in the thymus, as well as other secondary lymphoid tissues [26]. In the thymus, the process of negative selection plays a crucial role in establishing self-tolerance [27]. This process eliminates or suppresses developing T cells that recognize autoantigens expressed in the thymus, preventing the development of autoimmune diseases. This ensures that only T cells recognizing foreign antigens are allowed to exit the thymus and enter the bloodstream [28].

Autoantibodies targeting diverse retinal antigens have been identified in the bloodstream of patients, yet the exact process leading to the formation of these antibodies remains uncertain. The specific inquiry revolves around whether these antibodies play a role in the initial onset of retinal disease or if they emerge as a secondary occurrence during the progression of the disease. It has been suggested that ARAs targeting retinal proteins may be produced following three potential triggering events [29]:

1. Antitumor response: Malignant and benign cancers can elicit an immune response, as tumoral antigens exposed to antigen-presenting cells induce the production of ARAs against epitopes that cross-react with retinal proteins [30–32]. These retinopathies are termed CAR or MAR.
2. Anti-microbial infection: Similarity between proteins present in pathogens and the retina can lead to this reaction. An illustration of cross-reaction occurs in the glycolytic pathway, which plays crucial metabolic roles in both microbial and retinal cells [33].
3. Retinal injury: This mechanism may result from various factors, including trauma, inflammation, vascular disorders, and degenerative diseases. Causative mutations can induce cellular stress in photoreceptors, leading to their apoptosis and the generation of metabolic debris. Subsequently, this debris may contribute to autoimmunization.

The precise ARAs implicated in these conditions remain incompletely understood; however, various ARAs have been identified in separate research studies. ARAs targeting glycolytic enzymes, including aldolase, alpha-enolase, glyceraldehyde-3-phosphate dehydrogenase, and pyruvate kinase, were detected in the serum of individuals with retinal diseases. The elevated titers of these ARAs suggest a potential association with pathogenicity [33, 34]. Additional investigations into potential target proteins have yielded noteworthy results, including recoverin, rhodopsin, heat shock protein 27 (HSP27), one of Rab-related proteins (Rab6A), and carbonic anhydrase II (CA2) [35].

However, due to the scarcity of histopathological studies and animal models, the pathogenesis of AIR is mainly presumptive. It is thought that the development of antibodies against retinal proteins can trigger AIR. More specifically, they may be directed towards retinal-specific antigens, such as recoverin (23 kDa), which is a photoreceptor-specific calcium-binding protein or may be specific to non-ocular antigens, such as α-enolase (46 kDa), a glycolytic enzyme [2]. Although multiple antigens have been reported [36–38], recoverin and α-enolase constitute the most thoroughly investigated antigens and have been detected in both pAIR and npAIR. Recoverin, a 23 kDa calcium-binding protein found primarily in photoreceptor cells of the eye [1, 39], plays a crucial role in the inhibition of rhodopsin kinase that is responsible for the phosphorylation of rhodopsin. Upon binding with recoverin antibodies, degeneration of both rods and cones through apoptosis has been observed [40]. Enolase, a 46 kDa glycolytic enzyme, can be found in various tissues throughout the human body but is particularly abundant in neoplasms. It may be released during the turnover or removal of tumor cells [41, 42]. In the retina, it is detected in the cell membranes and cytoplasm of ganglion cells, Müllercells, rods, and cones. Patients with CAR, MAR, and npAIR have been found to possess antibodies targeting rod transducin [41]. Rod transducin is a guanine nucleotide-binding protein consisting of three subunits, which facilitates the connection between cGMP-phosphodiesterase and rhodopsin. This connection initiates the phototransduction cascade and causes hyperpolarization of the photoreceptor [41]. The genes responsible for encoding transducin subunits in rods (GNAT1) and cones (GNAT2) are distinct [43]. Mutations in the GNAT1 gene have been linked to congenital stationary night blindness [44, 45]. Abnormal expression of the transducin antigen occurs in cancer cells, and it is believed that autoantibodies targeting this antigen may disrupt signaling pathways, leading to changes in intracellular calcium levels and subsequent apoptosis [41]. Particularly, in npAIR, other consistently recognized antigens targeted by ARAs comprise arrestin (S-antigen) (48 kDa), carbonic anhydrase II (CA II) (30 kDa), interphotoreceptor binding protein (IRBP) (141 kDa). Associations with TULP1, neurofilament protein, heat shock protein-70, photoreceptor-cell-specific nuclear receptor (PNR), Müller-cell-specific antigen, transient receptor potential cation channel, subfamily M, member 1 (TRPM1), and other unknown proteins have also been reported [13, 46–51]. Ueno et al. [52] investigated in mice the role of autoantibodies against transient receptor potential melastatin 1 (TRPM1) protein in the dysfunction of ON bipolar cells in patients with paraneoplastic retinopathy (PR). They hypothesized that the anti-TRPM1 antibodies present in the serum of PR patients contribute to the degeneration of retinal ON bipolar cells. To test this, they injected PR patient serum, known to contain anti-TRPM1 antibodies, into mice and observed that the injected serum altered the electroretinograms (ERGs) of the mice, resembling the pattern seen in PR patients. By immunohistochemical analysis they confirmed the presence of anti-TRPM1 antibodies in the serum, specifically targeting bipolar cells in wild-type mice but not in TRPM1 knockout mice. By histological examination, they revealed apoptotic bipolar cells in the wild-type mice after 5 h, while no cell death occurred in the TRPM1 knockout mice. They observed that after 3 months, the inner nuclear layer was thinner, and ERG amplitudes remained reduced. Therefore, Ueno et al. [52] suggested that the serum of PR patients contains antibodies against TRPM1, leading to acute death of retinal ON bipolar cells in mice.

The exact role of ARAs in the npAIR pathogenesis is not fully understood, but it is believed that all types of AIR have an indistinguishable mechanism related to ARAs that are linked to retinal damage. A number of both in vitro and in vivo studies have shown that ARAs have a cytotoxic effect on retinal cells through various mechanisms such as caspase-associated apoptotic pathways etc. [38, 41, 53–55]**.** Indeed, Adamus [38] investigated the association between circulating antibodies targeting retinal proteins and retinal dysfunction in individuals with retinopathy. They observed that anti-recoverin antibodies found in patients with CAR are toxic to retinal cells, leading to apoptotic cell death of photoreceptor cells and subsequent degeneration of the photoreceptor cell layer leading to retinal cell death and vision loss. Another study by Adamus [54] asked the question of whether anti-recoverin antibodies in CAR patients' affect intracellular calcium levels, potentially causing cell apoptosis. The results showed that exposure to these antibodies increased intracellular calcium levels, which could be suppressed by a calcium channel blocker called nifedipine. Nifedipine also prevented changes in the levels of anti-apoptotic and pro-apoptotic proteins and reduced the release of cytochrome c and caspase 3 activation. They suggested that elevated intracellular calcium contributes to retinal dysfunction and degeneration in CAR, providing a basis for considering calcium blockers as a treatment option. Magrys et al. [55] investigated the role of autoantibodies against alpha-enolase, in visual loss and retinal degeneration in autoimmune and cancer-associated retinopathy. They found that these antibodies inhibited enolase's catalytic function, leading to ATP depletion, increased intracellular calcium levels, and apoptotic cell death. They observed that blockade of the calcium channels prevented the rise in intracellular calcium and induction of apoptosis. They suggested that autoantibodies against alpha-enolase disrupt glycolysis in retinal neurons, leading to their destruction and contributing to retinopathy. Lastly, Adamus et al. [41] studied the presence of anti-CAII autoantibodies in CAR and AIR without cancer. These antibodies impaired the catalytic function of CAII, resulting in decreased intracellular pH, increased intracellular calcium levels, and reduced retinal cell viability. This study suggested that anti-CAII autoantibodies have pathogenic effects on retinal cells, contributing to retinal degeneration. The above findings provide insights into the role of the immune system in retinal degeneration and may aid in developing better therapeutic strategies for autoimmune retinopathy.

Of particular interest are results from experimental studies that showed that the intravitreal injection of human IgG from MAR patients into the eyes of monkeys led to ERG waveforms similar to MAR, known as negative ERG (i.e., a reduced dark-adapted b-wave accompanied by a normal a-wave), which is indicative of the disruption of ON-bipolar cell signaling [56]. Subsequently, TRPM1, a member of the transient receptor potential cation channel subfamily M, which is expressed by melanocytes and ON-bipolar cells, has been identified as a crucial element in the functioning of ON-bipolar cells, and as the target of ARAs in MAR patients [46, 57]. However, it is yet to be defined whether ARAs are the actual cause of AIR pathogenesis or if they develop as a result of retinal degeneration. It has been speculated that the presence of tumor antigens (identical to those of the retina) in CAR and MAR stimulates the generation of ARAs, which have the potential to cross-react with retinal antigens [30, 51]. Likewise, the production of ARAs in npAIR could occur via molecular mimicry between retinal antigens and proteins present in bacteria or viruses. Alternatively, it is possible that the production of ARAs occurs due to exposure to retinal antigens following the retinal damage, and these ARAs may then contribute to the progression of the disease. Although some studies have indicated that ARAs play a crucial role in the development of pAIR, their mere presence is not sufficient to confirm the diagnosis of AIR [58]. Antigens targeting the retina have been detected in various conditions including healthy individuals [53], patients with systemic autoimmune disorders [59–61], uveitides, and other retinal pathologies [40, 62, 63]. Furthermore, in some patients exhibiting clinical symptoms and diagnostic findings of AIR, ARAs have not been detectable. Out of a group of 193 patients displaying clinical manifestations of AIR, such as painless vision loss, photopsia, and abnormal ERG, less than half (47.1%) tested positive for ARAs [53].

Despite extensive research, it is still unclear why some individuals with ARAs develop the disease, while others do not. ARAs can also be found in normal individuals, as the immune system may be exposed to self-antigens due to cell degradation. In fact, a study by Ko et al. [64] found ARAs in 42% of normal healthy controls. Moreover, these antibodies have been identified in several autoimmune and degenerative diseases, such as Behçet's disease, systemic lupus erythematosus (SLE), multiple sclerosis (MS), and age-related macular degeneration (AMD), although their significance is not yet fully understood [49].

In the dystrophic Royal College of Surgeons (RCS) rat model, it was observed that the presence of antigenic material released from deteriorating photoreceptor cells during retinal degeneration led to the generation of anti-retinal ARAs and T cells, exhibiting distinct activation trends [65]. Initially, there was a robust anti-photoreceptor antibody response, diminishing around day 40 in rats, followed by a resurgence in antibody production. This resurgence was triggered by renewed antigenic stimulation as more cells perished, persisting until the disappearance of photoreceptor cells when self-antigens released from dying cells became unavailable [65]. This implies that while ARAs may not instigate pathogenic processes, they can significantly influence their advancement. Furthermore, the adoptive transfer of anti-retinal ARAs from RCS rats with inherited retinal degeneration induced disruptions in the blood–retinal barrier, upregulated MCP-1 (CCL2) chemokine, and attracted macrophages/microglia into the retina. The increased migration of microglia correlated with elevated levels of photoreceptor apoptosis, impacting long-term photoreceptor survival [65]. It is probable that these early anti-retinal ARAs may contribute to cell death initially and later, to the progression of retinal degeneration by recruiting activated macrophages/microglia and generating new ARAs. Although ARAs are commonly detected in the circulation of individuals with vision loss, determining the primary mechanism of antibody formation is challenging. Evaluating cross-reactivity between cancer-retina and microbial-retinal antigens using tissue samples or purified antigens aids in identifying the source of antigenic stimulation. However, pinpointing ARAS generated due to retinal death poses a more formidable challenge. Regardless of their origin, anti-retinal ARAs tend to persist over time in circulation and are linked to stable or progressive vision loss [66]. Due to the prolonged nature of retinal autoimmunity, ARAs are likely to emerge before the manifestation of clinical symptoms, though testing non-symptomatic patients is not feasible. Such ARAs could serve as valuable predictive biomarkers for potential retinal disease and neoplasm development.

The 12 most well-characterized ARAs, whose pathogenic mechanisms have been more described, are listed in Table 1. Additional ARAs have been reported; however, their pathogenicity remains uncertain, and further studies are needed to confirm their clinical relevance.

**Table 1:** Antiretinal auto-antibodies [29–66]

Auto-antibody	Antigen	Molecular weight	Associated cell/tissues	Mechanism/effect
Anti-recoverin	Recoverin	23 kDa	Photoreceptor cells	Degeneration of rods and cones through apoptosis
Anti-α-enolase	α-enolase	46 kDa	Various retinal cells (including ganglion cells, Müller cells, rods, and cones), particularly abundant in neoplasms	Disrupts glycolysis, leading to ATP depletion, increased intracellular calcium, and apoptotic cell death
Anti-rod transducin	Rod transducin	38 kDa, 36 kDa, 10 kDa (three subunits)	Rod cells	Disrupts phototransduction, leading to alteration of intracellular calcium levels and retinal cell apoptosis and degeneration
Anti-arrestin (S-antigen)	Arrestin (S-antigen)	48 kDa	Retinal cells	Disruption of phototransduction Interference with rhodopsin deactivation Cellular stress Induction of apoptosis Retinal degeneration
Anti- CA II	CA II	30 kDa	Retinal cells	Impairment of enzymatic activity Disruption of pH regulation Increased intracellular calcium Induction of apoptosis and retinal degeneration
Anti-IRBP	IRBP	141 kDa	Retinal cells	Targeting IRBP Disruption of retinoid transport and therefore disrupting the visual cycle that is essential for phototransduction The presence of anti-IRBP antibodies can trigger an inflammatory response within the retina Photoreceptor degeneration Cell apoptosis Blood-retinal barrier disruption
Anti-TRPM1	TRPM1	182 kDa	ON bipolar cells	Causes degeneration of ON bipolar cells
Anti-TULP1	TULP1	64 kDa	Photoreceptor cells	Disruption of photoreceptor function Impaired protein trafficking Induction of inflammation Photoreceptor cell death
Anti-neurofilament protein	Neurofilament protein	60 kDa (L), 100 kDa (M), 200 kDa (H)	Neurons, including retinal ganglion cells	Disruption of structural integrity and interference with axonal transport Induction of inflammation Cellular dysfunction and death
Anti-heat shock protein-70	Heat shock protein-70	70 kDa	Retinal cells	Protein misfolding and stress response disruption Induction of inflammation Cellular apoptosis
Anti-PNR	Photoreceptor-cell-specific nuclear receptor	49 kDa	Photoreceptor cells	Gene expression disruption Impaired Photoreceptor function Induction of apoptosis
Anti-Müller-cell-specific antigen	Müller-cell-specific antigen	N/A	Müller cells	Disruption of Müller cell function Induction of inflammation Cellular dysfunction and death

ATP, adenosine triphosphate; CA II, Carbonic Anhydrase II; H, Heavy chain; IRBP, Interphotoreceptor Binding Protein; L, Light chain; M, Medium chain; PNR, Photoreceptor-Cell-Specific Nuclear Receptor; TRPM1, Transient Receptor Potential cation channel subfamily M member 1; TULP1, Tubby-Like Protein 1

To enhance our comprehension of AIR, future studies must investigate other potential factors (e.g., genetic and environmental factors) that may contribute to its pathogenesis. A relevant study [58] assessed 24 patients with npAIR and found a substantial correlation of npAIR with HLA-DRB1-03 and HLA-DRB1-15.

## Clinical manifestations

Remarkably, pAIR (CAR, MAR) and npAIR exhibit immunological and symptomatic heterogeneity. Individuals diagnosed with npAIR generally exhibit painless gradual loss of vision. This visual impairment is typically more severe than what may be anticipated based on their clinical presentation. A diffuse dysfunction of the photoreceptors in npAIR may cause visual symptoms such as photopsia, scotomas, dyschromatopsia, nyctalopia/photoaversion, and metamorphopsia [66, 67]. Even though the typical presentation of npAIR is asymmetrically bilateral, there have been several reported cases of unilateral AIR and suspected npAIR whose diagnosis was based on clinical evaluation and no antibody testing [68–70].

Furthermore, the clinical findings may vary depending on the severity and duration of the disease (Table 2) and even though in some cases ophthalmic fundus examination may appear unremarkable, in others signs of retinal degeneration may be evident. These signs may include vascular attenuation (narrowing of the blood vessels), cystoid macular edema (CME), atrophy of the outer retina and/or the RPE, and optic disc pallor. Deposits of pigment resembling bone spicules are not commonly observed. Electroretinogram (ERG) testing can confirm abnormalities in both scotopic and photopic responses, which may take on different configurations such as rod-cone, cone-rod, or electronegative patterns. Visual field testing may also reveal corresponding scotomas and constriction of the peripheral vision [1, 15, 66].

**Table 2:** Main clinical and diagnostic features in patients with autoimmune retinopathies

AIR type	Basic demographics	Clinical presentation	Diagnostic features	Prognostic indicators	Additional comments
npAIR	Usually affects middle-aged individuals (30–60 years) with a slightly higher incidence in females	Gradual bilateral vision loss Photoreceptor dysfunction symptoms	Asymmetric bilateral presentation Varying retinal signs (vascular attenuation, CMO, outer retina/RPE atrophy)	CMO occurrence inconsistent; EZ length loss associated with worse VA; More research needed for prognostic indicators	Unilateral cases reported FAF may reveal parafoveal ring
CAR	Typically seen in older adults (> 50 years), with a slightly higher incidence in males	Progressive bilateral vision loss Cone and rod dysfunction symptoms	Predominant cone dysfunction Colour vision and acuity worsening Symptoms worse than clinical signs	Retinal arteriole attenuation; Waxy optic disc pallor; Heterogeneous phenotype	Similar clinical phenotype to npAIR Frequently associated with anti-recoverin antibodies Rule out genetic retinal dystrophies or medical retinopathy
MAR	Most common in adults aged 40–70, with a higher frequency in males due to the male predominance of melanoma	Night blindness, visual field defects, photopsia Potential vitreous inflammation	Less heterogeneous presentation VA may vary Central and paracentral scotomas frequent	Optic disc pallor Vascular attenuation RPE changes Vitreous cells Accelerated progression with immunosuppressive agents	Immune response indicator to metastasis Potential diagnostic confusion with intermediate uveitis

AIR, autoimmune retinopathy; CAR, cancer-associated retinopathy; CMO, cystoid macular oedema; EZ, ellipsoid zone; MAR, melanoma-associated retinopathy; npAIR, non-paraneoplastic AIR; VA, visual acuity

Similarly to other retinal dystrophies, imaging with fundus autofluorescence (FAF) may reveal a parafoveal ring of increased autofluorescence [71], along with thinning of the outer nuclear layer and attenuation of the ellipsoid zone, as seen on optical coherence tomography (OCT). Additionally, there may be patchy areas of decreased autofluorescence peripherally. Fluorescein angiography (FA) usually does not show any significant abnormalities. Although mild leakage from the retinal blood vessels may be detected, this is typically less compared to cases with primary retinal vasculitis, whereas signs of intraocular inflammation are generally limited [14].

The occurrence of CME in npAIR is inconsistent. Ferreyra et al. [15] suggested that CME could be indicative of a distinct npAIR subtype, which is more responsive to immunosuppression. However, some patients with npAIR may experience persistent CME despite undergoing multiple treatments with immunomodulatory agents [72, 73]. Finn et al. [73] evaluated CME as a biomarker of disease progression in 16 npAIR patients (32 eyes). They observed that eyes with CME at both presentation and follow-up had a more rapid ellipsoid zone loss on foveal-centered spectral-domain (SD) OCT, as well as lower maximal a-wave and b-wave amplitudes on ERG compared to eyes without CME at either time point (*n* = 21). Moreover, eyes with CME had worse VA at presentation and the one-year follow-up. As expected, these eyes also had shorter ellipsoid zone length at presentation. Given that CME in these cases results from RPE dysfunction, it is reasonable to postulate that the extent of outer retinal damage would be more pronounced in cases presenting with CME. However, the results of these studies should be interpreted with caution due to the small and uneven sample sizes, as well as the lack of adjustment for potential confounding factors such as age, disease duration, and variations in treatment administration and response. Therefore, more research is necessary to determine whether CME, ellipsoid zone length, and other biomarkers may be useful as prognostic indicators in npAIR.

### CAR

**Fig. 1:**
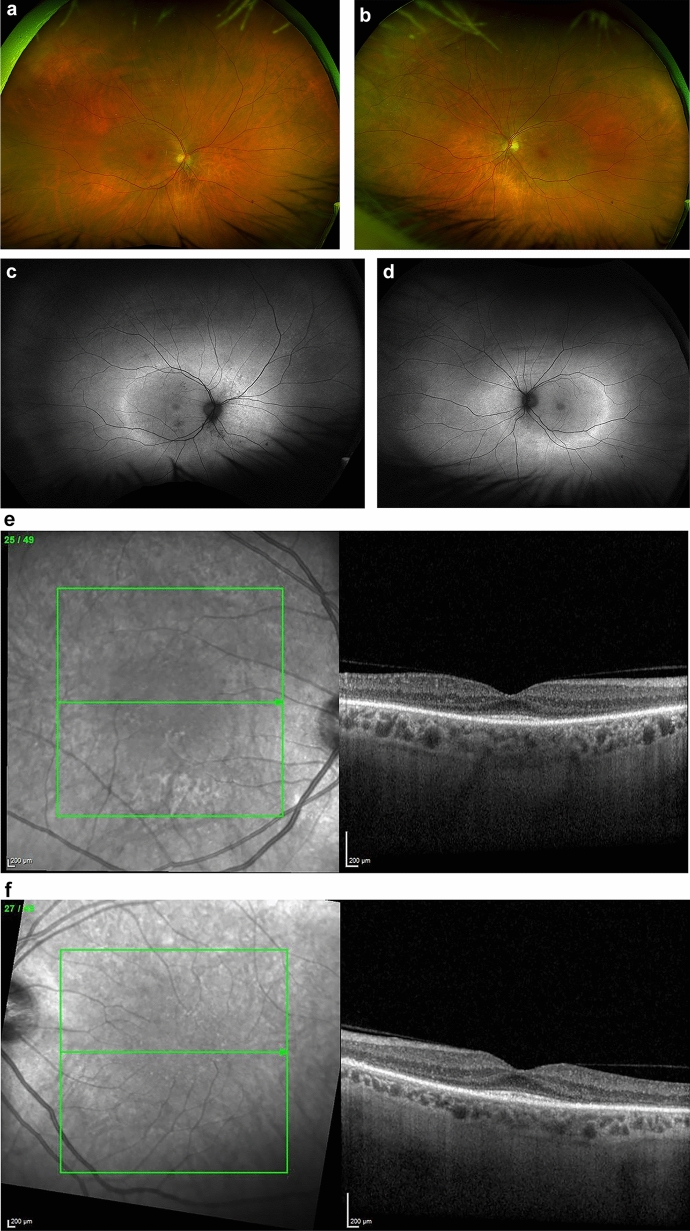

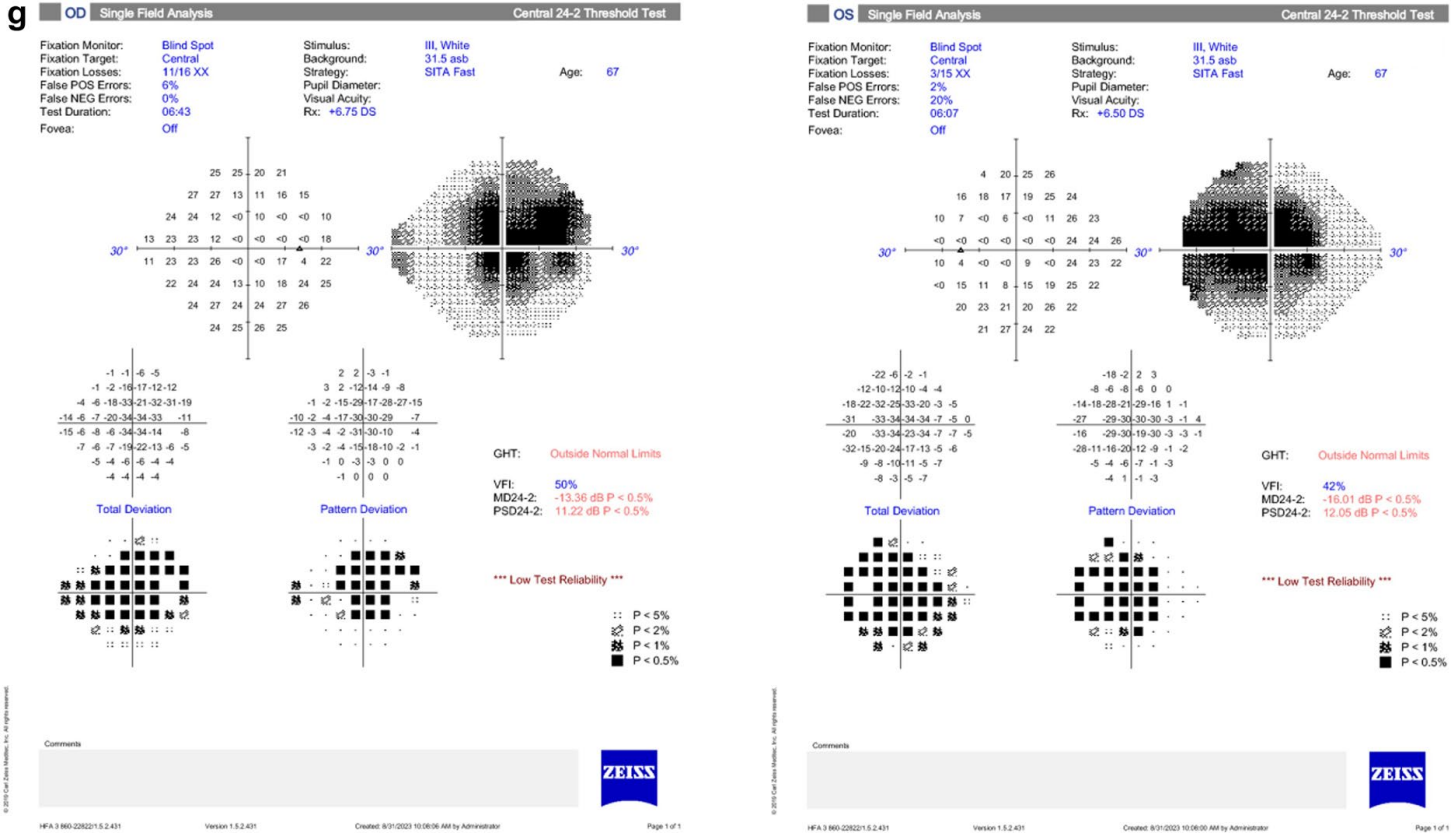
A 67-year-old female patient diagnosed with cancer-associated retinopathy showed Optos widefield color photos of the right (**a**) and left (**b**) eyes that could be deemed unremarkable. However, bilateral RPE changes, predominantly in the right eye, and a significant ring of hyperautofluorescence were evident in autofluorescence images (**c** right, **d** left). The hyperautofluorescent ring corresponds to a defect in the ellipsoid zone, as observed in the OCT scan (Heidelberg Spectralis EDI-OCT), revealing only a small central "island" of ellipsoid zone remaining in both eyes (**e** right, **f** left). The 24-2 Humphrey visual field test reveals bilateral nonspecific visual field defects (g). It is noteworthy to mention that electrodiagnostic tests revealed abnormalities in both eyes, with significantly attenuated mixed cone/rod and isolated rod ERG responses. These findings suggest retinal dysfunction, particularly affecting the rods. Moreover, occipital pattern VEPs exhibited severe degradation and broadening, pointing towards additional post-retinal dysfunction. EDI OCT, Enhanced depth imaging optical coherence tomography; ERG, Electroretinogram; RPE, Retinal pigment epithelium; VEPs, Visual evoked potentials

CAR is typically characterized by a progressive, occasionally asymmetric, bilateral vision loss that can lead to blindness over a period of days to years [66] (Fig. 1a–g). Predominant cone dysfunction is indicated by worsening color vision and acuity, along with symptoms of photosensitivity, glare, flickering or shimmering lights, and central scotoma. Impaired dark adaptation, night blindness, ring scotoma, and peripheral field defects are indicative of predominant rod dysfunction [66, 74]. The phenotype is heterogeneous, and simultaneous involvement of both rods and cones is common, especially in cases associated with anti-recoverin antibodies [16, 66]. The fundus often appears normal, and symptoms are generally worse than the clinical signs [66]. Retinal arteriole attenuation and waxy optic disc pallor may develop later in some cases [66], while iritis and vitritis have also been recorded in some cases [75]. Genetic retinal dystrophies or any medical etiology for retinopathy (e.g., pseudo-retinitis pigmentosa due to syphilis or drug toxicity) need to be ruled out as npAIR remains a diagnosis of exclusion. Genetic testing is essential in the differential diagnosis with inherited retinal diseases (IRDs) that may present with progressive photoreceptor dysfunction, visual field defects, and abnormal ERGs-features that overlap with npAIR. CAR has a similar clinical phenotype to npAIR, with similar heterogeneity [12, 66], but npAIR patients tend to be younger compared to those with CAR [16, 17]. Family or medical history of autoimmune disease is frequent in these patients, but no other risk factors or disease associations have been recognized. Interestingly, concerning familial autoimmunity, the occurrence of various autoimmune disorders within the same family, has been overlooked in comparison with the clustering of a single autoimmune condition among relatives. The study of Cárdenas-Roldán et al. [76] confirmed the existence of familial autoimmunity across all studied conditions, with autoimmune thyroid disease being the most frequently recorded, followed by systemic lupus erythematosus and rheumatoid arthritis. Their findings underline the significance of further research to delineate shared mechanisms underlying autoimmunity.

### MAR

MAR typically manifests as night blindness, visual field defects, and photopsia. While its presentation is less heterogeneous than that of CAR, atypical presentations have also been recorded [77] In a relevant study that included 62 MAR cases, 82% of these patients presented with VA of 6/18 or better, and central and paracentral scotomas were frequent [78]. The fundoscopic examination was unremarkable in some patients, while others had optic disc pallor, vascular attenuation, RPE changes, and vitreous cells. Limited follow-up data showed that approximately half of the patients had unilateral or bilateral moderate to severe loss of vision at the last follow-up [78]. Based on our clinical experience, MAR may exhibit a greater manifestation of vitreous inflammation, potentially leading to diagnostic confusion with intermediate uveitis. It is pertinent to emphasize that the presence of MAR serves as an indicator of an immune response to metastasis, and consequently, the administration of immunosuppressive agents in these patients may accelerate the progression of melanoma.

In cases with paraneoplastic optic neuropathy, the optic disc may appear normal, edematous, or atrophic, along with the presence of vitreous cells and retinal vascular leakage. Patients typically experience bilateral subacute visual loss, which progresses painlessly over a few days [79].

## Diagnostic approach

**Fig. 2:**
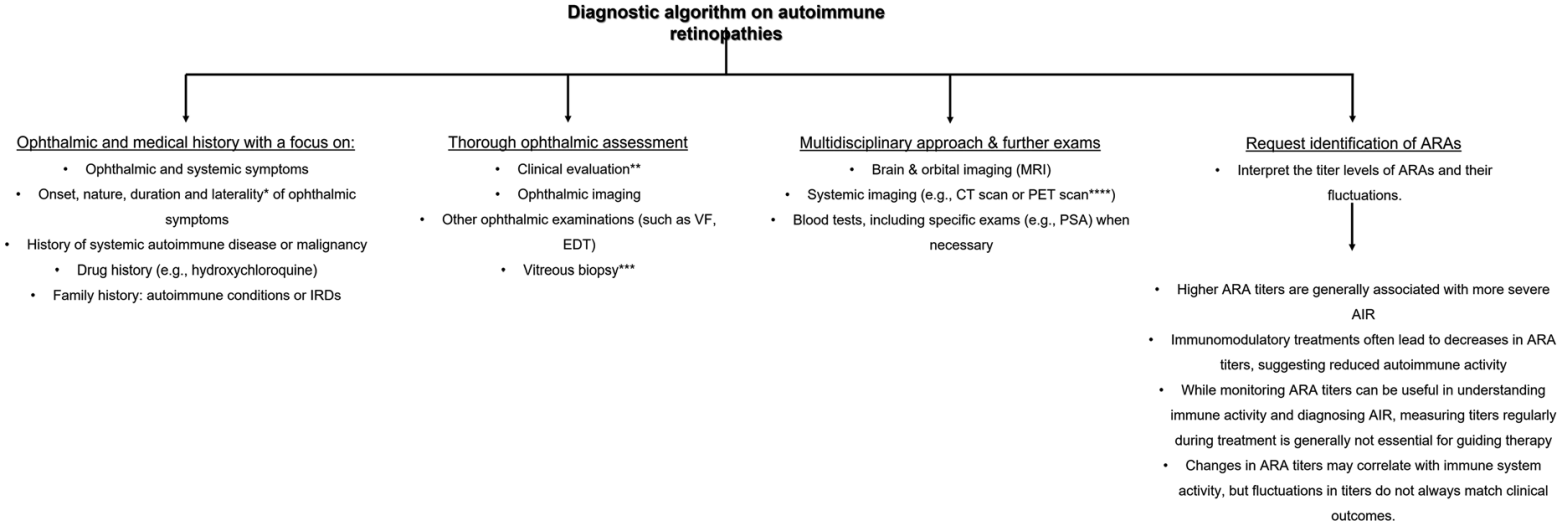
A diagnostic algorithm for autoimmune retinopathies. *The typical presentation is asymmetrically bilateral; however unilateral cases have been reported. **The clinical findings may vary depending on the severity and duration of the disease. In some cases, the examination may appear entirely normal. ***Vitreous biopsy can be crucial in ruling out intraocular lymphoma. ****Especially when malignancy is suspected [The major cancer associations observed included lung, breast, melanoma, hematological (lymphomas, leukemias, and myelomas), gynecological, prostate, and colon]. AIR, autoimmune retinopathy; ARAs, anti-retinal antibodies; CT, computed tomography; EDT, electrodiagnostic tests; MDT, multidisciplinary team; MRI, magnetic resonance imaging; PSA, prostatic specific antigen; PET, positron emission tomography; VF, visual fields

A consensus statement from 2016 established essential diagnostic criteria, including the absence of other identifiable causes of visual function abnormalities, abnormal electroretinogram (ERG) results, the presence of serum ARAs, and the absence of significant intraocular inflammation [19]. Additional supportive criteria include personal/family history of autoimmune disease, signs/symptoms of photoreceptor dysfunction, and rapid onset of vision changes [2]**.** Diagnostic evaluations could include fundus autofluorescence, ERG, serum ARA testing (preferably through a two-tiered assay), and comprehensive oncological workup. The ophthalmic work-up could also include perimetry, FAF, FA, and microperimetry to rule out other causes of retinopathy [47, 80, 81] (Table 3) (Fig. 2).

**Table 3:** Available diagnostic modalities in autoimmune retinopathies

Diagnostic Tool	Purpose	Findings in AIR	Special Considerations
Fundus autofluorescence (FAF) [62, 63, 81]	Identify distinct patterns of damage to the RPE; Track the advancement of AIR	Widespread or granular stippled hyper-autofluorescence, anomalies in FAF patterns	Crucial for monitoring AIR progression; recommended in imaging protocol
SD-OCT [18, 47, 64, 80–86]	Provide objective measurements of retinal damage; Facilitate diagnosis of AIR	Loss of outer retinal complex, disruption of EZ, ONL thinning	Can aid in early diagnosis; CMO frequently observed; EZ disruption common; delays in diagnosis associated with EZ extent reduction
Visual field testing [2]	Detect constriction, peripheral losses, scotomas, enlargement of the blind spot	Non-specific findings aiding in diagnosis	Helpful in establishing the diagnosis despite non-specificity
Electrophysiology (ERG, mf-ERG, EOG) [3, 12, 16, 18, 50, 66, 87–90]	Provide quantifiable and objective measurements of electrical signals; Differentiate retinal disorders	Cone dysfunction, negative ERG in MAR, abnormalities in a- and b-waves Abnormal ERG results in all eyes at presentation; abnormal findings in mf ERG	Full-field ERG, mf ERG, and EOG used; Delay in b-wave, reduced amplitudes common; "electronegative" ERG observed; global retinal damage in some cases CAR linked to cone response delay; "Electronegative" ERG not pathognomonic; central or global cone dysfunction more frequent than rod dysfunction in anti-enolase-associated retinopathy

AIR, autoimmune retinopathy; CAR, cancer-associated retinopathy; CD, cluster of differentiation; CME, cystoid macular oedema; EOG, electrooculogram; ERG, electroretinogram; EZ, ellipsoid zone; IL, interleukin; IVIG, intravenous immunoglobulin; MAR, melanoma-associated retinopathy; mf-ERG, multifocal ERG; npAIR, non-paraneoplastic AIR; RPE, retinal pigment epithelium; ONL, outer nuclear layer; SD-OCT, spectral domain optical coherence tomography; TNF, tumour necrosis factor

### Fundus autofluorescence

**Fig. 3:**
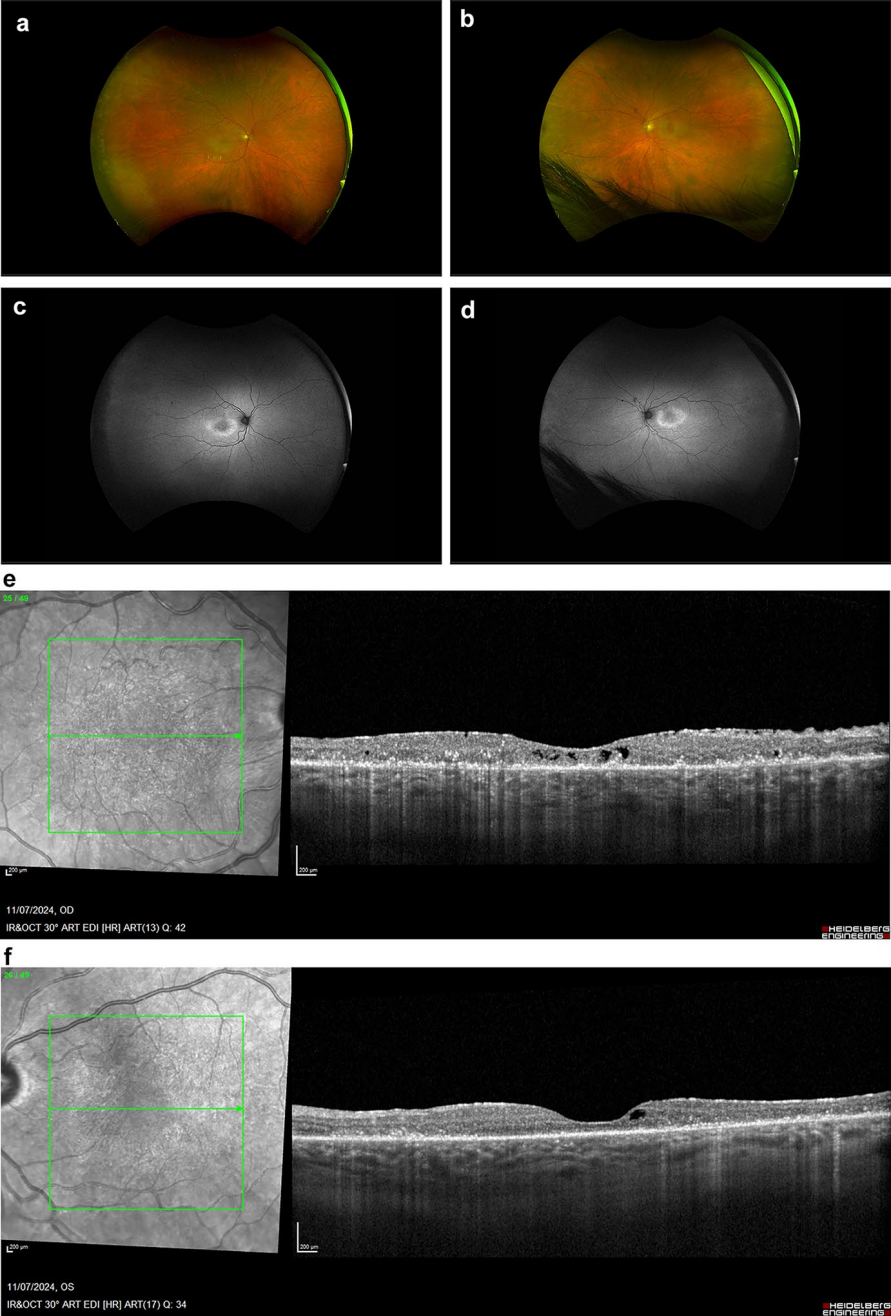

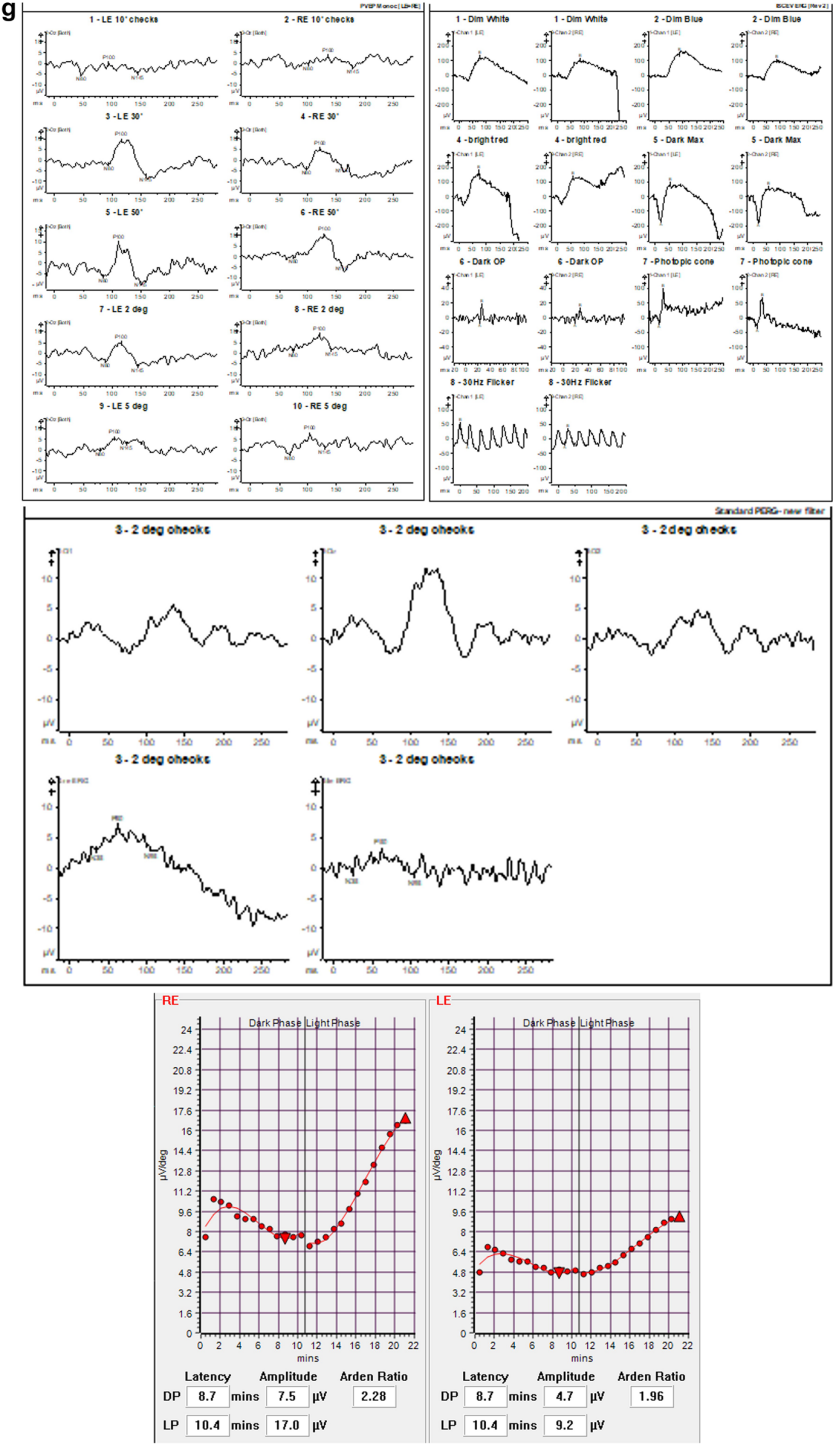
75-year-old female with history of acute myeloid leukemia with myelodysplastic syndrome-related changes. Approximately 16 months post allogeneic peripheral blood stem cell transplant using Fludarabine, Treosulfan, and Alemtuzumab. Optos widefield fundus color images indicate macular pigmentary changes in the right eye (**a**) and left eye (**b**), corresponding to the hyper-autofluorescent ring observed in the fundus autofluorescence images (**c** right eye, **d** left eye). Spectralis SD-OCT (Heidelberg Engineering, Heidelberg, Germany) demonstrates bilateral thinning of the outer nuclear layer and attenuation of the ellipsoid zone (**e** right eye, **f** left eye), along with an epiretinal membrane and intraretinal cystic spaces. **g** The results of the electrodiagnostic tests. Electrooculogram (EOGs) was normal. The mixed cone/rod flash electroretinogram (ERGs) and isolated cone ERGs were of borderline amplitudes. Rod ERGs were significantly reduced below the lower limits of normal amplitude. Pattern ERGs were of borderline amplitudes. Occipital pattern Visual Evoked Potential (VEPs) were degraded and broadened. These results were consistent with retinal dysfunction involving rods more than cones. Based on the above findings, a diagnosis of cancer-associated retinopathy was established, but the antiretinal autoantibodies were negative

Imaging with FAF can capture the distinct patterns of damage to the RPE (Fig. 3a–d). Anomalies in FAF (Fundus Autofluorescence) patterns are prevalent among most patients and are identified by either a widespread or granular appearance of stippled hyper-autofluorescence across the posterior pole, particularly in the macular and peripapillary areas. This increase in autofluorescence is attributed to the abnormal accumulation of lipofuscin derivatives in the metabolically hyperactive RPE [62]. Additionally, disruptions to the inner-outer segment junction and a thinning of the outer nuclear and photoreceptor layers can also lead to hyper-autofluorescence, thereby increasing the visibility of the underlying RPE autofluorescent signal [63]. Even in the peripheral regions, large areas of hyperautofluorescence may be observed, creating a demarcation between the affected and unaffected retinal areas. Notably, the scope of these lesions did not exhibit a discernible correlation with best corrected visual acuity (BCVA). In contrast to wide-angle fundus photography, wide-angle fundus FAF proved to be more effective and informative in delineating the extent of these lesions. Nevertheless, both of these imaging modalities did not reveal any observable changes during subsequent follow-up visits for any of the patients, irrespective of the progression status. Overall, due to its ability to identify even the most subtle structural alterations that may be challenging to discern with ophthalmoscopy alone, FAF imaging emerges as a crucial tool in tracking the advancement of AIR in patients. It is recommended to incorporate FAF imaging into the standard imaging protocol for monitoring this condition. Finally, FAF is extremely useful in discerning from other retinal disorders such as hydroxychloroquine retinopathy [82].

### Spectral-domain optical coherence tomography

The increasing availability of SD-OCT in clinical practice, which provides higher spatial resolution than time-domain OCT, may provide further insights into the cytoarchitectural integrity of these patients (Fig. 3e, f) [2]. As demonstrated in previous studies, the use of SD-OCT can provide objective measurements of retinal damage and facilitate the diagnosis of AIR [47, 81, 83, 84]. Retinal abnormalities detected with SD-OCT [e.g., loss of the outer retinal complex or disruption of the ellipsoid zone (EZ)], can be indicative of a diagnosis of AIR before the results of the laboratory tests and electroretinography (ERG) become available [47, 81, 83, 84]. According to Lima et al. [47], EZ loss was discovered in four patients with AIR, which corresponded to a hyper-autofluorescent ring. Abazari et al. [83] demonstrated that OCT can identify outer retinal pathology and help in the selection of patients that require further investigation. In the same study, seven out of eight patients with AIR had outer retinal abnormalities, such as loss of the photoreceptor layer or disruption of the photoreceptor outer and inner segment junction [83]. Similarly, Sepah et al. [84] showed that AIR patients had substantial loss of retinal tissue, specifically of the photoreceptor layer. Finally, a more recent study by Khanna et al. [18], demonstrated that the attenuation of the outer nuclear layer (ONL) and EZ was the most frequent retinal damage pattern on SD-OCT. On the contrary, the foveal region showed relatively preserved outer retinal elements.

No existing reports indicate a correlation between treatment and an increase in the anatomical strength or extent of EZ [18, 47, 81, 83, 84]. Considering the potential for renewal in the outer segments of photoreceptors, it is conceivable that the initiation and duration of treatment in these studies were insufficient to observe any anatomical changes on SD-OCT. The collective data published consistently emphasize that a prolonged time to diagnosis is associated with a decline in visual acuity and a more substantial reduction in the sub-foveal EZ extent based on SD-OCT measurements over time [18]. Moreover, delays in diagnosis may indicate a point beyond which regeneration of photoreceptor outer segments becomes unattainable.

The EZ has been reported to recover after intravitreal injection of dexamethasone in a patient with acute zonal occult outer retinopathy (AZOOR) [85] and after treatment with difluprednate in a patient with npAIR [86]. In a case series where EZ regeneration was observed, less severe npAIR was present, and an intact ELM overlying the EZ suggested a shared pathway with other similar maculopathies [18]. These observations suggest that in rare cases where recovery of the EZ occurs, various factors, such as time to diagnosis, treatment type and duration, and management of related systemic disorders, may play a role in the recovery process.

In a recent study [80] OCT imaging revealed focal or diffuse depletion of the outer retina, affecting both the ELM and the EZ in seven out of eight patients. When occurring bilaterally, the outer retinal involvement exhibited asymmetry in three out of six patients. Eyes exhibiting RPE changes along with ELM and EZ losses demonstrated a more significant decline in BCVA compared to those with RPE changes alone. Among the sixteen eyes examined, nine displayed disorganized RPE, characterized by focal losses sharply delineated over the underlying Bruch’s membrane. In 18.7% of cases (three eyes), there was progressive deterioration of RPE/Bruch’s membrane disorganization observed during the follow-up period. The aforementioned studies have demonstrated that EZ disruption and loss on OCT are common findings in AIR, which can be useful in diagnosing and monitoring the disease progression.

The frequency of CME in SD-OCT reports is variable, ranging from 24 to 50% [64]. In the case series published by Khanna et al. [18] 66% of the eyes had CME at presentation. Interestingly, the same study showed that ARA testing revealed a higher incidence of anti-enolase antibodies and staining in the photoreceptor layer in eyes with CME. The presence of CME at initial presentation or its subsequent development indicates a more severe and aggressive disease with decreased ERG a- and b-wave amplitudes compared to eyes without CME [73]. Eyes with CME at presentation also have a greater rate of EZ length loss between baseline and final follow-up [18].

### Fundus fluorescein angiography

Fluorescein fundus angiography (FFA) is an extremely useful imaging modality in suspected AIR cases, as it can provide further insights visualizing various features including retinal vascular abnormalities, leakage, and macular oedema. Particularly, leakage from retinal vessels arises from the increased vascular permeability and is suggestive of autoimmune or inflammatory processes. Apart from these AIR-specific features, FFA can demonstrate hypofluorescent areas of RPE atrophy or degeneration, which is associated with retinal damage. Furthermore, RPE disruption can lead to areas of hyperfluorescence or window defect. Evaluating all these imaging findings can contribute to defining the degree of retinal damage and also to differentiate AIR from other retinal pathologies, such as vascular disorders or IRDs [1, 2, 5, 13, 19].

### Indocyanine green angiography

The use of indocyanine Green Angiography (ICGA) can play a pivotal role assessing the RPE and deeper choroidal layers. In AIRs, ICGA can detect early choroidal involvement that may not be obvious on FFA, particularly when there is inflammation of the choroidal vessels. Furthermore, it can identify choroidal leakage or filling defects, which are characteristic in conditions such as inflammatory choroidal neovascularization (CNV). Moreover, as it can detect changes in choroidal circulation and the onset of CNV, suggesting either progression or treatment failure. Therefore, ICGA can be useful in monitoring response to treatment. Both FFA and ICGA can provide substantial information and help in distinguishing AIR from other retinal disorders, highlighting the inflammatory nature of AIRs [1, 5, 13, 19].

### Visual field testing

Although non-specific, perimetry may detect constriction, peripheral losses, scotomas, enlargement of the blind spot, and pericentral losses that can aid in establishing the diagnosis [2].

### Electrophysiology

Electrophysiological tests can serve as a valuable tool in the diagnosis of suspected AIR by providing a quantifiable and objective measurement of the electrical signals produced by the eye, optic pathways, and visual cortex [87] (Fig. 3g). These tests can help differentiate between disorders that affect different retinal layers and cell types by revealing different electroretinographic patterns. Certain electroretinographic patterns may also be associated with specific clinical entities of autoimmune retinopathies, such as CAR, MAR or npAIR, or different ARAs. However, there is a lack of comprehensive electrophysiological data for patients with AIR in the existing literature, which makes it difficult to draw definitive conclusions. Therefore, despite the variety of electrophysiological characteristics, none of them can provide a conclusive diagnosis for the condition.

The multifocal ERG can pinpoint the topographic location of the disease within the retina. In addition, the electrooculogram (EOG) can measure the retinal pigment epithelium layer's integrity [87]. The leading edge of the full-field ERG a-wave response, which is triggered by a bright flash stimulus, is thought to indicate the isolated response of the photoreceptors [87]. In contrast, the b-wave is a composite of contributions from multiple neural elements, with the bipolar cells contributing significantly [87]. The photopic, cone-driven b-wave, however, represents a combination of activity from on- and off-bipolar cells. Disorders that specifically affect the photoreceptors can potentially impact both the a-wave and b-wave responses of the ERG. Meanwhile, diseases that affect bipolar cells result in an absent b-wave and an a-wave that is recorded as a negative deflection, which gradually returns to the baseline but does exceed it. Oscillatory potentials (OPs) are believed to be indicative of activity in a complex feedback circuit that involves bipolar, amacrine, and inner plexiform cells [87].

CAR is often linked to ARAs and affects cone responses in ERG [3, 50]. Studies suggest that patients with CAR may exhibit a delay in b-wave, reduced amplitudes of both a- and b-waves, reduced b-wave, and an electronegative ERG [88]. MAR is characterized by a negative waveform on a standardized full-field ERG, caused by a decrease in b-wave amplitudes [78]. A complete loss of full-field ERG was reported in one patient with npAIR while others showed selective b-wave loss [12]. According to a study on AIR, full-field ERG demonstrated abnormal results in all eyes at presentation, while 73% of eyes showed abnormal findings on mf ERG [18].

However, the "electronegative" ERG, described above, is not pathognomonic and can also be observed in various other pathologies of the inner retina, such as congenital stationary night blindness. Therefore, it's essential to rule out these other conditions before making a diagnosis [89]. In their report, Thirkill et al. [90] observed similar loss of rod and cone amplitudes along with normal or prolonged implicit times in 10 CAR patients with antibodies against recoverin. Similarly, a case series of 39 patients with npAIR and CAR, associated with anti-rod transducin, reported electroretinography results for two-thirds of the cases, with all except one patient having abnormal results. These abnormalities were characterized by heterogeneous patterns, including reduced scotopic responses and irregular photopic cone and 30-Hz flicker responses, as well as prolonged rod and cone implicit times. Additionally, the ERG was extinguished in several eyes, which indicated global retinal damage [16]. According to Weleber et al. [66] central or global cone dysfunction was observed more frequently than rod dysfunction in a case series of 12 patients with anti-enolase-associated retinopathy, four of whom had cancer.

### Systemic assessment and screening for neoplasms

In cases of AIR, multidisciplinary approach is mandated, and it is recommended that an oncologist determines the need for further tests such as endoscopy, brain MRI, CT scan (of chest, abdomen, and pelvis), positron emission tomography-computed tomography, and gender-appropriate investigations such as prostate examinations or mammograms [33, 91–93]. It is crucial to distinguish between paraneoplastic and non-paraneoplastic subtypes because of their significant implications. Both types of retinopathies share similar symptoms such as vision loss, nyctalopia, photopsia, and scotomas, but pAIR tends to exhibit a more rapid decline. Small cell carcinoma of the lung is commonly associated with CAR, while cutaneous melanoma is typically associated with MAR. Therefore, an examination and investigation can be focused on these frequently encountered entities [39]. CAR may occur before the diagnosis of cancer, whereas MAR typically appears after the diagnosis of metastatic melanoma [19]. Benign tumors (e.g., oncocytoma) can also trigger the production of antiretinal antibodies, resulting in npAIR [93]. It is worth noting that benign tumors are frequently discovered unintentionally, and further research is necessary to determine a definitive causal connection [19].

### Identification of anti-retinal antibodies (ARAs)

According to several authors, the diagnosis of AIR requires the presence of ARAs [13, 15, 67]. However, as already highlighted above [19], these autoantibodies can be found in both healthy individuals and those with various ocular and systemic diseases. Proving their pathogenicity in different disease states is challenging and requires rigorous scientific evidence. The pathogenicity of ARAs probably depends on various parameters such as host factors, epitope-specificity, and the retinal microenvironment. In patients with suspected AIR, at least 17 ARAs have been found; however, physicians should differentiate those truly harmful from those that are not. The presence of ARAs in confirmed cases of AIR, their correlation with clinical symptoms, and their capacity to cause retinal damage in experimental models can all be used to assess their pathogenicity. Some ARAs might not directly contribute to the progression of the disease, but rather serve as secondary markers. [58].

Patients diagnosed with CAR, MAR, and npAIR exhibit ARAs primarily targeting intracellular proteins, with only a limited number directed at membrane proteins found in various retinal cell types [51, 94–97]. Among the initial ARAs linked to CAR are those targeting recoverin, a calcium-binding protein crucial for visual phototransduction [4, 30, 98]. Initially, seropositivity for anti-recoverin ARAs was observed exclusively in patients with small cell carcinoma of the lung [90, 99]. However, subsequent investigations revealed associations between anti-recoverin ARAs and various malignancies [100]. Despite recoverin being considered a key biomarker for CAR syndrome, the presence of anti-recoverin ARAs is infrequent, with only approximately 5% of CAR patients possessing such antibodies [35, 101]. It is important to note that the absence of ARAs against recoverin does not rule out a diagnosis of paraneoplastic syndrome. Currently, over 30 different antigens in the retina have been identified in association with vision loss [17, 102–104].

Presumed targets in the context of retinal degeneration are the photoreceptor cells located in the outer layer of the retina. Among these targets is recoverin, a protein found in photoreceptor cells, alongside various other photoreceptor antigens, including arrestin, guanylate cyclase-activating protein, transducin-α and transducin-β, TULP1, Rab6, rhodopsin, and IRBP [46, 52, 94, 95, 105–107]. However, patients with CAR exhibit ARAs in their sera that not only interact with antigens in photoreceptor cells but also with bipolar and ganglion cells within the retina [35, 99, 108, 109]. In the case of npAIR, ARAs targeting glycolytic enzymes, such as enolase, aldolase, glyceraldehyde-3-phosphate dehydrogenase, and pyruvate kinase M2, are prevalent in patients’ sera [33]. Additionally, antibodies against heat shock proteins, specifically HSP27 and HSP65, have been identified in patients with CAR and npAIR [35, 110, 111]. Notably, CAII emerges as a significant target protein in prostate cancer-associated CAR and npAIR [16].

In the context of MAR, dysfunction of retinal ON bipolar cells is linked to TRPM1, a membrane autoantigen [46, 51, 52, 97]. TRPM1 is also present in melanocytes [51, 97]. The epitope of the TRPM1 autoantibodies is situated in the short intracellular domain within the amino-terminal region of the TRPM1 sequence [46, 97]. The generation of TRPM1 ARAs is postulated to result from abnormal TRPM1 polypeptides encoded by an alternative mRNA splice variant expressed by malignant melanocytes [97]. However, these ARAs are uncommon, being found in less than 5% of MAR patients. More frequently, ARAs against proteins involved in phototransduction are identified, likely due to the expression of rhodopsin, transducin, cyclic guanosine 3′,5′-monophosphate phosphodiesterase 6, guanylyl cyclase 1, recoverin, and arrestin in human melanoma cells in vitro [112]. Indeed, MAR patients exhibit AAbs against transducin, rhodopsin, arrestin, and IRBP, as well as α-enolase, CAII, myelin basic protein, mitofilin, and titin [31, 107, 113–115]. In recent years, reports have emerged of a MAR-like retinopathy accompanied by detachments of the RPE and neurosensory retina. This clinical presentation is referred to as paraneoplastic vitelliform retinopathy, and in some instances, three additional proteins, in addition to the usual anti-enolase-α, anti-bestrophin, and anti-peroxiredoxin, were detected in the serum [116–118].

Patients with AIR, who do not report previous visual issues but experience a sudden onset of symptoms like photopsias, night blindness, scotomata, and visual field loss, were found to possess ARAs with similar specificities [14, 119]. ARAs against recoverin, α-enolase, aldolase, and CAII, along with ARAs targeting HSP27, HSP60 and CRMP2, were identified in AIR patients [35, 111, 120, 121]. More recently, Rujkorakarn et al. [122] conducted a retrospective cross-sectional study to examine the correlation between changes in ARAs titers and numbers with vision outcomes in patients with AIR. The study included 31 eyes from 16 patients who underwent anti-retinal antibody testing at least twice during follow-up. The results, analyzed in terms of visual acuity, subjective vision loss, visual field, and electroretinography outcomes, did not show any significant differences based on ARA titers or numbers. The study suggests that alterations in ARA titers or numbers may not be associated with changes in vision outcomes. The findings indicate that repeated ARA testing after the diagnosis of AIR and the detection of an ARA may be unnecessary.

Finally, AIR may also arise as a secondary complication of other conditions such as retinitis pigmentosa, birdshot retinopathy, or AZOOR. Retinitis pigmentosa patients with cystoid macular edema often exhibit antibodies against CAII [123]. While a high incidence of ARAs has been observed in AZOOR patients, it remains unclear whether this condition is mediated by autoimmune mechanisms [124, 125].

### Available laboratory techniques

Three distinct laboratory methods have been outlined for the detection of serum antiretinal antibodies (ARA), namely immunohistochemistry (IHC), Western blotting (WB), and enzyme-linked immunosorbent assay (ELISA), with IHC and WB being more prevalent. However, none of these methods can be considered conclusive. Each technique presents both advantages and drawbacks [13]. For instance, WB relies on the protein size for antibody identification, making it technically challenging and lacking in specificity. Consequently, it is recommended to employ two techniques simultaneously to enhance both sensitivity and specificity [14].

Thus, while some ARAs have been proven to be pathogenic through both in vitro and in vivo experimental studies, other retinal autoantibodies have not been studied as extensively. Shimazaki et al. [126] used Western blot techniques to demonstrate that most of the normal serum samples showed some level of anti-retinal reactivity, with 33% displaying reactivity against one to two protein bands and 22% exhibiting reactivity against five or more bands; notably, women's serum tended to show reactivity with three or more protein bands compared to men. Approximately 33% of the samples showed reactivity against one to two protein bands, while approximately 22% exhibited reactivity against five or more distinct bands. Considering the multitude of potential ARAs and the fact that even normal serum can have noticeable ARA activity, it is crucial to establish the pathogenicity of the autoantibodies suspected of causing AIR. Various authors have expressed concerns regarding the uncertainty surrounding the pathogenicity of ARAs [1, 2, 13, 36, 86, 127].

Ultimately, demonstrating the pathogenicity of these autoantibodies requires conducting basic scientific experimentation. Although cell culture studies provide limited data suggesting the pathogenicity of autoantibodies that target carbonic anhydrase II, no basic scientific studies have been published that examine the potential retinal toxicity of anti-TULP1 antibodies [16]. Given the current uncertainties surrounding the pathogenicity of ARAs in the field of AIR, conclusive scientific evidence, including animal studies, is necessary to investigate the potential pathogenicity of these antibodies.

## Diagnostic criteria

Different authors have suggested diagnostic criteria for AIR, but there is currently no international consensus on those [13, 15, 67, 119]. In contrast, international consensus has been established on diagnostic criteria for other immune-mediated eye diseases, such as Behçet’s disease or ocular sarcoidosis [128]. It would be advantageous to have comparable international consensus and standardization for AIR.

## Differential diagnosis

The differential diagnosis of AIR encompasses a range of conditions such as white dot syndrome (WDS) spectrum disorders (e.g., AZOOR), retinal degenerative disorders [e.g., retinitis pigmentosa and cone-rod dystrophy], and non-infectious and infectious uveitic entities [2, 5, 19]. AZOOR can produce comparable symptoms, visual field defects, and ERG findings, and is typically bilateral but can be asymmetrical. The majority of patients with AZOOR either remain stable or experience partial recovery without treatment. In contrast, Multiple Evanescent White Dot Syndrome (MEWDS), despite exhibiting similar symptoms, is a mostly unilateral retinopathy defined by an afferent pupillary defect, optic nerve swelling, and spontaneous recovery, making it more easily distinguishable from AIR. Both AZOOR and MEWDS may present with an enlarged blind spot-on visual field. Furthermore, most eyes affected by AZOOR may exhibit striking FAF features, with well-delineated areas of hypo-autofluorescence, which have not been reported in cases of AIR [129, 130].

### Ruling out genetic disorders

In several cases, differentiating IRDs from npAIR can be challenging, and therefore genetic testing should be requested. For instance, Cone-rod dystrophy and retinitis pigmentosa (RP) are linked to specific genetic mutations. Detecting a pathogenic variant in a known IRD gene can be diagnostic and rule out an autoimmune process [131]. On the other hand, progressive visual loss and an abnormal ERG are more suggestive of an autoimmune disorder, such as npAIR, if genetic testing does not reveal a causative mutation. Moreover, it must be taken into consideration that the retinal dysfunction in some individuals may be a result of both an autoimmune reaction and an underlying genetic predisposition. In such cases, genetic testing can help define whether an autoimmune disorder is superimposed or an IRD is the primary etiology. Considering the potential benefit of prompt immunomodulatory treatment in npAIR, while confirmed IRDs usually do not respond to immunosuppressive therapy, this differentiation is pivotal for decision-making. If IRD is suspected based on clinical manifestations and family history, meticulous genetic testing must be conducted. If serologic and clinical features are more suggestive of autoimmunity and genetic results are still obscure, immunosuppressive therapy can be considered. To ensure an accurate diagnosis and improve patient care, a multidisciplinary approach among ophthalmologists, geneticists, and immunologists is mandated [1, 2, 5, 13, 19].

## Therapeutic approach

The timely diagnosis and treatment of AIR are crucial to prevent irreversible damage to retinal cells caused by this rapidly progressive systemic disease. Early diagnosis has a high visual and systemic prognostic significance and can even help detect metastasis and precede the diagnosis of cancer in patients with pAIR [132–135]. A multi-disciplinary approach involving oncology, dermatology, rheumatology, and radiology may be necessary for complete evaluation and management.

Currently, there is no standard treatment protocol for AIRs. However, various therapeutic options have been described in the literature (Table 4). These include the following:

1. Immunosuppression with topical (intravitreal, sub-tenon, or depot) and/or systemic corticosteroids
2. Immunomodulators (e.g., azathioprine, cyclosporine, infliximab, mycophenolate mofetil)
3. Biologic agents such as monoclonal antibodies (e.g., rituximab, tocilizumab, alemtuzumab, ipilimumab, sarilumab)
4. Others such as antioxidant vitamins, plasmapheresis, intravenous immunoglobulin (IVIG), and carbonic anhydrase inhibitors

**Table 4:** Available treatments for autoimmune retinopathies

Treatment type	Examples	Mechanism of action	Effectiveness	Special considerations
Corticosteroids [69, 78, 119, 139–142]	Intravitreal, sub-tenon, or depot corticosteroids (e.g., triamcinolone)	Anti-inflammatory; minimize tumour load	Variable response; may avoid negative side effects with brief trial	Consider intravitreal triamcinolone before systemic steroids
Immunomodulators [72, 73, 78, 136, 143–147]	Azathioprine, cyclosporine, mycophenolate mofetil, methotrexate	Long-term immunosuppression	Variable response; may be considered for patients ineligible for surgery	Triple therapy regimen studied; anti-IL-6 receptor antibodies for refractory CMO
Biologic agents [10, 72, 128, 150–166]	Rituximab, infliximab, adalimumab, tocilizumab, alemtuzumab, ipilimumab, sarilumab	Target specific pathways (e.g., CD20, IL-6)	Positive response reported in CAR and npAIR; rituximab may be viable for npAIR	TNF-α inhibitors not commonly used due to infection concerns
IVIG and plasmapheresis [14, 36, 76, 119, 173]	IVIG, plasmapheresis	Competitive inhibition of autoantibodies; removal of harmful contents	Variable response: better outcomes when initiated early	IVIG may have limited efficacy; conflicting data on plasmapheresis
Cellular therapies [174–179]	Autologous stem cell transplant	Unclear; potential immune modulation	Limited evidence; questionable effectiveness	Uncertain efficacy in AIR; reported positive response in one case

AIR, autoimmune retinopathy; CAR, cancer associated retinopathy; CD, cluster of differentiation; CME, cystoid macular oedema; IL, interleukin; IVIG, Intravenous immunoglobulin; npAIR, non-paraneoplastic AIR; TNF, tumour necrosis factor

### Corticosteroids

Upon confirming a diagnosis of pAIR, the suggested course of action involves initially minimizing the tumor load through the application of radiotherapy, chemotherapy, and/or surgical interventions. [78, 133, 136]. In all instances of AIR, it is recommended to address any underlying systemic disease before initiating treatment. Subsequently, patients can be introduced to steroids (either local or systemic) and/or antimetabolites/T-cell inhibitors as the primary or secondary treatment options. [14, 15, 119, 137, 138].

It has been advised to conduct a brief treatment trial using intravitreal or sub-tenon triamcinolone, involving two injections spread over 8 months with a dosage range of 40-80mg, before starting systemic steroids (60-80mg prednisone per os daily), as a means of verifying the diagnosis and avoiding the negative side effects of treatment [69, 119, 139]. Intravenous methylprednisolone has shown better outcomes than oral prednisone and has been used to initiate treatment [76, 140, 141]. A case study has shown that repeated serial intravitreal triamcinolone injections can lead to complete restoration of retinal anatomy on OCT and improved vision in patients with CAR [139]. An additional clinical case has shown enhancements in visual acuity (VA), visual field, and retinal function in a patient with MAR. This improvement was achieved through the use of a bilateral intravitreal sustained-release fluocinolone acetonide implant, without concurrent systemic therapy [142]. Given that AIR is an autoimmune condition, the initial recommendation for treatment was an extended duration, ranging from 4 months to 1 year or beyond. This extended period aims at both stabilizing the condition and restoring lost visual function [119]. However, there is still debate on the potential benefits of this treatment [15, 66, 127], and a prospective randomized placebo-controlled clinical trial could provide important insights on this topic [14].

### Immunomodulation

A retrospective study reviewed the use of a triple therapy regimen for long-term immunosuppression. The treatment regimen involved administering cyclosporine (100 mg/day), azathioprine (100 mg/day), and prednisone (20–40 mg/day) to a cohort of 30 patients, consisting of 24 with npAIR and 6 with CAR. The duration of treatment varied from 3 to 89 months, with three patients also receiving intravenous immunoglobulin (IVIG) [15]. The study observed a treatment response in 70% of all patients, indicating that 54% of npAIR patients without CME and 73% of npAIR patients with CME exhibited a positive response to the treatment. However, the study had limitations such as its retrospective nature and the absence of pre-defined treatment protocols, leading to non-comparable baseline characteristics among patients. Recently, two case reports detailed the complete resolution of refractory CME in npAIR patients through the use of anti-interleukin (IL)-6 receptor antibodies. The first report employed five tocilizumab infusions at a dose of 8 mg/kg every 4 weeks [72, 73], while the second report utilized four subcutaneous sarilumab injections at a dose of 200 mg every 2 weeks. For MAR resistant to steroids, radiotherapy and cytoreductive surgeries were found to be beneficial [78]. Immunomodulatory treatment is recommended for patients who are ineligible for surgical intervention [136]. In a case of melanoma relapse unresponsive to surgery and chemotherapy, the antagonist antibody ipilimumab, targeting cytotoxic T-lymphocyte antigen-4, demonstrated effectiveness [143].

There are some smaller case series and individual case reports that provide evidence for the effectiveness of cyclosporine, mycophenolate, and azathioprine in treating npAIR [144–147]. However, since there are only a few nonresponsive patients, publication bias likely exists [66, 121, 148]. Notably, there is no record of the successful use of methotrexate in treating npAIR [149, 150].

### Biologic agents

Rituximab is a chimeric monoclonal antibody that targets CD20, which is present on the surface of B-cells at most stages of differentiation. Its main mechanism of action is believed to be the depletion of B-cells via apoptotic signaling, as well as complement- and antibody-mediated cytotoxicity [151]. Furthermore, rituximab also focuses on a particular subset of CD3 + CD20 + T-cells that are inclined towards generating proinflammatory cytokines [152]. In recent years, several studies have underlined the potential effectiveness of rituximab in both CAR and npAIR [150, 153–161]. These studies have described satisfying functional and anatomical outcomes, and overall positive response to rituximab treatment. Moreover, treatment with rituximab and concomitant immunosuppressive agents (such as mycophenolate, cyclophosphamide, IVIG, bortezomib, and topical steroids) demonstrated stable or improved visual outcomes in 77% of eyes in one retrospective case series of 16 AIR patients (including 1 MAR, 6 CAR, and 9 npAIR) [159]. Every six months, two distinct loading regimens were utilized: either two doses of 1000 mg every alternate week or a loading dose of 375 mg/m^2^ per week for 4 weeks. Similarly, Boudreault et al. [160] evaluated the efficacy of rituximab in four patients with npAIR and found that rituximab contributed to the stabilization of the progression of retinal dysfunction. Another retrospective analysis of four npAIR individuals who were treated with rituximab with/without combination treatment showed that VA was stabilized in two-thirds of patients, whereas an overall improvement in visual fields or ERG parameters was reported in 75% of patients [161]. Additionally, alemtuzumab (administered at a dose of 30 mg IV three times a week for four months) has also been reported as potentially beneficial in a CAR patient [162].

TNF-α plays a crucial role in regulating cellular survival, inflammation, and autoimmunity [163]. However, TNF-α inhibitors such as etanercept, infliximab, certolizumab, adalimumab, and golimumab have not been used in pAIR due to concerns about the potential increased risk of infections and secondary malignancies [164, 165]. Only infliximab and adalimumab have been used in the context of npAIR. Infliximab was effective in treating npAIR with CME in a 44-year-old female patient [15]. Adalimumab was not effective after cyclosporine and azathioprine had failed [156], while another case worsened after infliximab and mycophenolate mofetil [160]. However, both patients responded positively to rituximab and remained stable for several years. These findings suggest that rituximab may be a viable treatment option for npAIR.

IL-6 is a cytokine that plays a role in inflammation, B-cell differentiation, and IgG production [166]. Tocilizumab and sarilumab are two biologic agents that target the IL-6 pathway and have been used to treat npAIR. One 46-year-old woman who had been clinically progressing for 3 years despite various treatments, including immunosuppression with mycophenolate mofetil and rituximab, showed significant improvement after treatment with tocilizumab, with a near-complete resolution of macular edema and improvement in visual acuity [73]. Similarly, a 29-year-old woman showed improvement in visual acuity, macular edema, and ERG amplitudes after treatment with sarilumab, and the macular edema remained resolved after 6 months of biweekly injections [10].

Bortezomib is a proteasome inhibitor used for hematologic malignancies that induce cell cycle arrest and apoptosis [128]. It has only been used in conjunction with rituximab, often with other immunosuppressive therapy, for npAIR [120, 122]. One patient received rituximab with bortezomib after failing other treatments but did not experience improvement and remained stable after treatment cessation [150].

It is strongly recommended that the administration of all the mentioned agents be carried out in collaboration with oncologists to ensure proper coordination. Additionally, it is essential to consistently rule out infections before initiating this type of treatment in patients [167].

### Intravenous immunoglobulin (IVIG) and plasmapheresis

IVIG and plasmapheresis are potential treatment options, especially if administered before irreversible neuronal degeneration occurs [14, 36, 76, 119, 168]. IVIG is a purified serum of polyclonal IgG that is pooled from more than ten thousand donors. Although the exact mechanism of how it works is not fully understood, it is believed that IVIG can shorten the half-life of pathogenic autoantibodies and competitively inhibit their binding. It may also contain anti-idiotype antibodies that target proinflammatory mediators, such as cytokines, activated complement proteins, and cell-adhesion molecules [169]. When initiated early, IVIG has been shown to result in better clinical response, particularly for CNS-related syndromes. A patient with CAR had sustained improvement in VA and visual fields with a 5-day course of IVIG at 400 mg/kg/day [141]. In MAR, published data have documented improved visual outcomes with a dose of 100 g of IVIG given over 2 consecutive days, followed by subsequent doses every 4–8 weeks [78]. However, there is no standard dose recommended due to limited literature and evidence supporting its use as a monotherapy. Ferreyra et al. [15] described three npAIR patients who received IVIG treatment but did not respond to the therapy. The first patient, a 42-year-old woman, had already undergone treatment with several immunosuppressive medications, including subtenon methylprednisolone and prednisone, cyclosporine, and azathioprine, without any improvement. The second patient, a 51-year-old woman, had to discontinue all oral medications due to adverse effects and only received 5 months of immunosuppressive therapy, including cyclosporine, azathioprine, prednisone, and intravitreal triamcinolone acetonide. Similarly, the third patient, who received IVIG before mycophenolate mofetil and intramuscular methylprednisolone acetate, showed no evidence of improvement. Abazari et al. [83] described three cases of npAIR that were unresponsive to oral prednisone for at least 4 weeks and were subsequently treated with IVIG. Of the three patients, only one with normal full-field ERG responses at baseline showed an improvement in visual field testing and multifocal ERG. The other two patients who had lost full-field ERG responses only reported subjective improvement in photopsias.

Plasmapheresis, also known as therapeutic plasma exchange, involves the removal of blood plasma, including harmful contents such as autoantibodies, immune complexes, and monoclonal proteins [170]. The removed plasma is typically replaced with the patient’s own plasma or a colloidal solution like 5% albumin. Plasmapheresis may be helpful in MAR by removing ARAs, immune complexes, and cytokines [78]. Its use is suggested for non-responders or individuals who do not meet the criteria for alternative therapies [153]. However, data on the benefits of plasmapheresis for npAIR are conflicting [14]. A 31-year-old female with a past medical history of myasthenia gravis had developed npAIR despite prior treatment with IVIG, prednisone, and mycophenolate mofetil. Her visual acuity dropped significantly (hand movements) over 2 years, and she was started on weekly plasmapheresis for over 1 year, which ameliorated her symptoms related to myasthenia gravis but did not improve her visual function [171]. Moreover, a 41-year-old male with npAIR associated with a renal oncocytoma showed negligible response to a 5-day course of intravenous methylprednisolone and five courses of plasmapheresis. However, only after the oncocytoma resection, he experienced a progressive improvement in visual function over 9 months [93]. On the other hand, a 35-year-old man with AIR and optic neuropathy had gradually worsening vision for 4 years but showed stabilization of visual acuity after undergoing 6 courses of plasma exchange [172]. Another 60-year-old lady diagnosed with npAIR associated with autoimmune cerebellar ataxia had also maintained visual function over a period of 21 months after receiving plasmapheresis and IVIG. However, the specifics of the treatment and testing were not mentioned [173].

### Cellular therapies

Cellular therapy has emerged as a potential treatment for degenerative retinal diseases like AMD and RP. Various cell sources, including human fetal and adult retina as well as pluripotent human stem cells, have been explored for this purpose [174–177]. However, it is unlikely that this approach would benefit AIR patients. The reviewed research brings attention to a potential issue that could arise when transplanting large quantities of retinal-antigen-laden cells into the eyes of individuals with AMD or RP. The transplantation process often results in the immediate death of a significant number of cells, leading to a sudden release of retinal photoreceptor antigens [174–177]. This release has the potential to trigger an autoimmune retinopathy-like response. Studies on animal models of outer retinal degeneration have shown progressive compromise of the blood-retinal barrier [178] and chronic T cell-mediated rejection of non-autologous photoreceptor transplants [179]. While pigment epithelial cells in the subretinal space may have immune modulatory mechanisms, similar to those observed in anterior chamber-associated immune deviation, it remains uncertain whether these mechanisms can provide sufficient immune protection to transplanted photoreceptor progenitor cells in the subretinal space.

A 47-year-old female patient with breast cancer was diagnosed with AIR and optic neuropathy suffered from vision loss for 7 months. Despite receiving prednisone, methotrexate, plasmapheresis, and IVIG, her visual function continued to deteriorate over 3 years. She underwent a non-myeloablative unmanipulated autologous stem cell transplant following immune ablation using cyclophosphamide 200 mg/kg and alemtuzumab 20 mg. Her visual acuity and visual field improved and remained stable 2 years post-transplant [162]. However, the treatment effects may also be attributed to the agents used for immune ablation, making this report interesting yet questionable [91].

### Carbonic anhydrase inhibitors

Currently, there are not adequate data on the role of carbonic anhydrase inhibitors in the treatment of AIR. Their use is limited to the management of patients with elevated intraocular pressure and persistent cystoid macular oedema as adjuvant therapy [180].

### The role of local therapy

Local therapy in AIR aims to address complications that may arise as a result of the inflammatory process, such as macular oedema, neovascularization, and vascular leakage. The most common therapeutic options involve intravitreal steroid injections and anti-VEGF agents, which may suppress the inflammatory activity and also improve the visual potential. Other therapeutic modalities, such as laser photocoagulation or vitreoretinal surgery are less common but can prove helpful in selected cases. The choice of local treatment is individualized and is usually defined by the severity of the disease and how the patient responds to systemic immunosuppression. A multi-modal approach combining systemic and local therapeutic tools is typically the most effective way to treat AIR and minimize the loss of vision [1, 2, 5, 13, 14, 19].

## Other therapeutic considerations

Heng et al. [181] conducted a retrospective analysis of 3 patients with advanced cutaneous melanoma who developed AIR following immunotherapy. In case 1, photopsia and nyctalopia were observed, and electroretinographic findings were consistent with melanoma-associated retinopathy 1 week after initiating ipilimumab/nivolumab immunotherapy. Case 2 experienced severe bilateral visual field loss alongside anti-retinal and anti-optic nerve antibodies while on maintenance nivolumab immunotherapy. Case 3 developed decreased visual acuity due to acute exudative polymorphous vitelliform maculopathy within 2 weeks of initiating ipilimumab/nivolumab immunotherapy. All patients had additional extraocular immune-related adverse events, and serological testing confirmed the presence of anti-retinal antibodies. This study demonstrates that immune checkpoint inhibition can induce the development of AIR with various clinical manifestations in patients with advanced cutaneous melanoma. It emphasizes the importance of close monitoring in cutaneous melanoma patients undergoing immunotherapy, especially if they experience new visual symptoms, regardless of funduscopic changes. Additionally, the study suggests the potential role of screening patients before initiating immunotherapy.

Systemic cancer therapies (i.e., chemotherapy, immunotherapy, and targeted therapies) can introduce new ophthalmic risks and complications, such as retinal toxicity, worsening of retinal oedema, or increased ocular inflammation. Therefore, a coordinated multidisciplinary approach among the Oncologist and the Ophthalmologist is essential to provide a tailored approach to each patient, ensuring effective cancer control while preserving retinal health.

## Prognosis

The visual prognosis for most cases of antiretinal antibody syndrome is poor, and there is little evidence of significant spontaneous visual improvement. In some cases, the antiretinal antibody type may be associated with the final visual outcome. CAR and npAIR patients with anti-enolase-associated retinopathy typically experience vision loss no worse than 20/300, whereas those with anti-recoverin antibodies may progress to having no perception of light (NPL). CAR and MAR patients often have a high mortality rate due to underlying malignancy. However, studies suggest that CAR patients who are seropositive for anti-recoverin antibodies may have improved survival compared to those who are seronegative [143, 144], possibly due to peripheral activation of recoverin-specific anti-tumor cytotoxic T-lymphocytes [143]. It is unclear whether immunosuppression therapy has a negative impact on the life expectancy of these patients. Cyclodestructive surgery with adjuvant immunotherapy has been found to reduce the tumor load in MAR patients, leading to improved visual function in several cases [60].

## Overcoming current limitations

### An urgent need for standardization and validation

Different laboratory methods have been reported for identifying circulating ARAs, such as immunohistochemistry (IHC), Western blot, and enzyme-linked immunosorbent assay (ELISA), with each technique presenting its own set of strengths and weaknesses [39].

When detecting ARAs, incorporating appropriate controls (i.e., positive controls, negative controls, loading controls, replicates, and standard curves) is essential, regardless of the technique employed [20, 32]. Failure to implement rigorous controls can result in false-positive and false-negative results. For instance, in Western blotting, using appropriate negative controls is necessary to avoid false positives against anti-recoverin and anti-α-enolase, the two most commonly reported ARAs in AIR [13, 36]. Concerns have previously been raised regarding the lack of proper control reporting in the ARA literature [13, 42, 49].

ARA testing is available commercially in various laboratories [52], with some having Clinical Laboratory Improvement Amendments (CLIA) certification for this service [16]. However, it's important to note that CLIA approval only ensures good laboratory practices and does not guarantee the standardization and validation of the test being offered. The lack of standardization among different laboratories offering this testing has resulted in inconsistent test results and poor agreement between laboratories [182]. To address this, some experts recommend sending patient samples to at least two different laboratories to ensure more accurate results [36].

Validation of ARA testing is another aspect that needs substantial improvement, particularly in establishing its correlation with clinical disease. To achieve this, the sensitivity, specificity, positive predictive value, and negative predictive value of ARA testing need to be determined. However, none of these parameters have been established for any ARA test [2]. Additionally, several bands are usually detected on Western blot analysis in most AIR patients [15, 119]. However, not all of these bands necessarily represent pathogenic ARAs. Thus, validated criteria based on the number, intensity, molecular weight, and isotype of these bands are needed to assist clinicians in evaluating the clinical relevance of these results. A similar approach is used in the diagnosis of Lyme disease using Western blot, where certain specific bands must be present for the diagnosis of Lyme to be confirmed [183]. However, such validated criteria are currently not available for ARA testing.

### The lack of prospective randomized trials

Several previous review articles have thoroughly discussed the treatment of AIR, with a consensus among authors that underlying malignancies should be addressed before proceeding with immunomodulatory therapy [1, 2, 13, 15, 36, 67, 119]. As already mentioned above, the latter approach has primarily involved the use of systemic or local corticosteroids, intravenous immunoglobulins, plasmapheresis, and systemic immunosuppression, among other strategies [1]. There are only a few small studies that have investigated the treatment of AIR, and they have reported inconsistent results. While some studies have reported improvement with treatment, others have reported worsening despite treatment. Additionally, reports that utilize these immunomodulatory approaches are limited to retrospective case reports and case series [2, 15, 50, 66]. A study involving the largest patient cohort to date included 30 patients who were treated with immunosuppression (both local and systemic), showing that 70% of patients experienced improvement [15]. However, this study [15] had limitations due to its retrospective nature, lack of a predefined treatment regimen with various treatments, varying patient follow-up, and differing clinical presentations of AIR. Considering the suggested antibody-mediated nature of AIR, targeting B cells with a therapeutic agent that reduces systemic antibody levels may be a reasonable approach. Rituximab, a monoclonal antibody (anti-CD20) that targets B cells, has been demonstrated to be a successful treatment of AIR in a few case reports [153, 155, 184]. However, the difficulty in applying treatment findings for AIR from the available literature is based on retrospective reviews. As of now, there are no prospective, placebo-controlled, and/or randomized studies evaluating the treatment of AIR. Furthermore, there are no reliable markers available to assess treatment effects [15]. Until such data become available, the treatment options for AIR will remain uncertain.

## Conclusions

The diagnosis and management of AIR continue to pose challenges. Although different diagnostic criteria for AIR have been suggested, there is currently no international consensus on these criteria. While the identification of circulating ARAs is deemed by several authors as a crucial diagnostic criterion for AIR, there is still no consensus on the pathogenicity of certain antibodies. Numerous studies have demonstrated the presence of diverse retinal autoantigens that specifically engage with ARAs in patients affected by AIRs. Certain ARAs, particularly those targeting cancer or microbial cross-reactive antigens, are likely to play a central role in causing retinal degeneration, while others may contribute to disease progression and facilitate damage to retinal cells. An argument has been made that ARAs result from retinal demise rather than directly causing retinopathy, functioning more as an epiphenomenon. Consequently, the apoptosis of photoreceptors initiated by other mechanisms (such as hereditary factors) generates significant debris, containing high concentrations of antigens released from dying outer segments. This process may lead to autoimmunization and increased permeability of the blood–retinal barrier. There is a lack of randomized controlled trials for AIR treatment, and an evidence-based treatment strategy has yet to be established. Although several therapeutic methods have been proposed for treating AIR, there is no universally accepted standard protocol for its treatment. In light of these difficulties, ophthalmologists should take into account AIR when treating patients who exhibit progressive visual impairment that cannot be explained, especially when those patients have autoimmune diseases or underlying cancers. To rule out other possible causes of retinopathy, a methodical diagnostic approach is necessary, involving multimodal imaging, electrophysiology, and serological testing for ARAs. A high index of suspicion can lead to early diagnosis and prompt treatment reducing the risk of permanent damage to retinal cells. For individualized treatment, a multidisciplinary team comprising rheumatologists, neurologists, oncologists, and ophthalmologists is essential after AIR is diagnosed. Although response rates vary, immunosuppressive therapy is still the cornerstone of care. Corticosteroids, intravenous immunoglobulin, or steroid-sparing immunomodulatory agents are frequently used. Because AIR is heterogeneous, tracking treatment response is still difficult. Periodically, functional visual assessments, ERGs, and serial multimodal imaging should be carried out to evaluate the course of the disease and the effectiveness of treatment. Alternative treatment approaches, like targeted biologic therapies, may be taken into consideration in situations where immunotherapy is ineffective or inappropriate. Despite recent advancements in understanding AIR, uncertainties remain. The enigmatic nature of AIR diagnosis and management will persist as long as these uncertainties are not resolved.

## Data Availability

No datasets were generated or analysed during the current study.
